# Secondary Bile Acids and Tumorigenesis in Colorectal Cancer

**DOI:** 10.3389/fonc.2022.813745

**Published:** 2022-04-28

**Authors:** Yujing Liu, Shengan Zhang, Wenjun Zhou, Dan Hu, Hanchen Xu, Guang Ji

**Affiliations:** ^1^Institute of Digestive Diseases, Longhua Hospital, Shanghai University of Traditional Chinese Medicine, Shanghai, China; ^2^Department of Internal Medicine of Chinese Medicine, Shanghai Pudong New Area Hospital of Traditional Chinese Medicine, Shanghai, China

**Keywords:** colorectal cancer, bile acids, tumorigenesis, intestinal flora, high-fat diet

## Abstract

Colorectal cancer (CRC) is one of the most common and deadly cancers in the world and is a typical inflammatory tumor. In recent years, the incidence of CRC has been increasing year by year. There is evidence that the intake of high-fat diet and overweight are associated with the incidence of CRC, among which bile acids play a key role in the pathogenesis of the disease. Studies on the relationship between bile acid metabolism and the occurrence of CRC have gradually become a hot topic, improving the understanding of metabolic factors in the etiology of colorectal cancer. Meanwhile, intestinal flora also plays an important role in the occurrence and development of CRC In this review, the classification of bile acids and their role in promoting the occurrence of CRC are discussed, and we highlights how a high-fat diet affects bile acid metabolism and destroys the integrity of the intestinal barrier and the effects of gut bacteria.

## Introduction

Bile acid is a metabolite of cholesterol in the liver. Bile acid can promote the absorption of fat and fat-soluble vitamins in the intestine and plays an important role in lipid metabolism in the liver (1). In addition, in the liver, bile acids can regulate sugar metabolism, lipid metabolism, and bile acid homeostasis through activation of farnesol X receptors (FXRs) (2, 3), which are related to the occurrence of several diseases.

In the human body, bile acids are synthesized in the liver cells by oxidation of cholesterol. They can be divided into free bile acids and conjugated bile acid according to their structure. Bile acids can also be divided into PBAs and secondary bile acids according to their source. The production of PBAs occurs mainly through the classical and alternative pathways (4). The classical pathway is initiated by cholesterol 7α-hydroxylase (CYP7A1) (5), which mainly produces cholic acid (CA) and chenodeoxycholic acid (CDCA). In addition, sterol‐12α‐hydroxylase (CYP8B1) and mitochondrial sterol‐27‐hydroxylase (CYP27A1) play key roles in the classical pathway (6). The alternative pathway is initiated by CYP27A1 and mainly produces CDCA (5). In addition, oxysterol‐7a‐hydroxylase (CYP7B1) plays a major role in the alternative pathway (5). Subsequently, in hepatocytes, CA and CDCA are conjugated with glycine and taurine to form taurocholic acid (TCA), glycine cholic acid (GCA), taurine chenodeoxycholic acid (TCDCA), and glycine chenodeoxylate oxycholic acid (GCDCA) (7). After these acids are excreted into the intestine through the biliary system, they are uncoupled into CA and CDCA by the bile salt hydrolases (BSH) of the intestinal bacterial and then converted into secondary bile acids through 7α-dehydroxylation, including deoxycholic acid (DCA) and lithocholic acid (LCA). Ursodeoxycholic acid (UDCA), on the other hand, was first isolated from the bile of Chinese black bears. Under physiological conditions, the content in human body is very small, less than 4% of the total bile. UDCA is a 7-β isomer formed by deoxycholic acid (CDC) through the action of intestinal bacteria (8). Finally, most bile acids are reabsorbed at the end of the ileum and transported back to the liver through the portal vein system. A small volume of bile acids is excreted through the feces. The process of bile acid circulating in the intestine and liver is called enterohepatic circulation (9).

## Bile Acids and CRC

**Figure 1:**
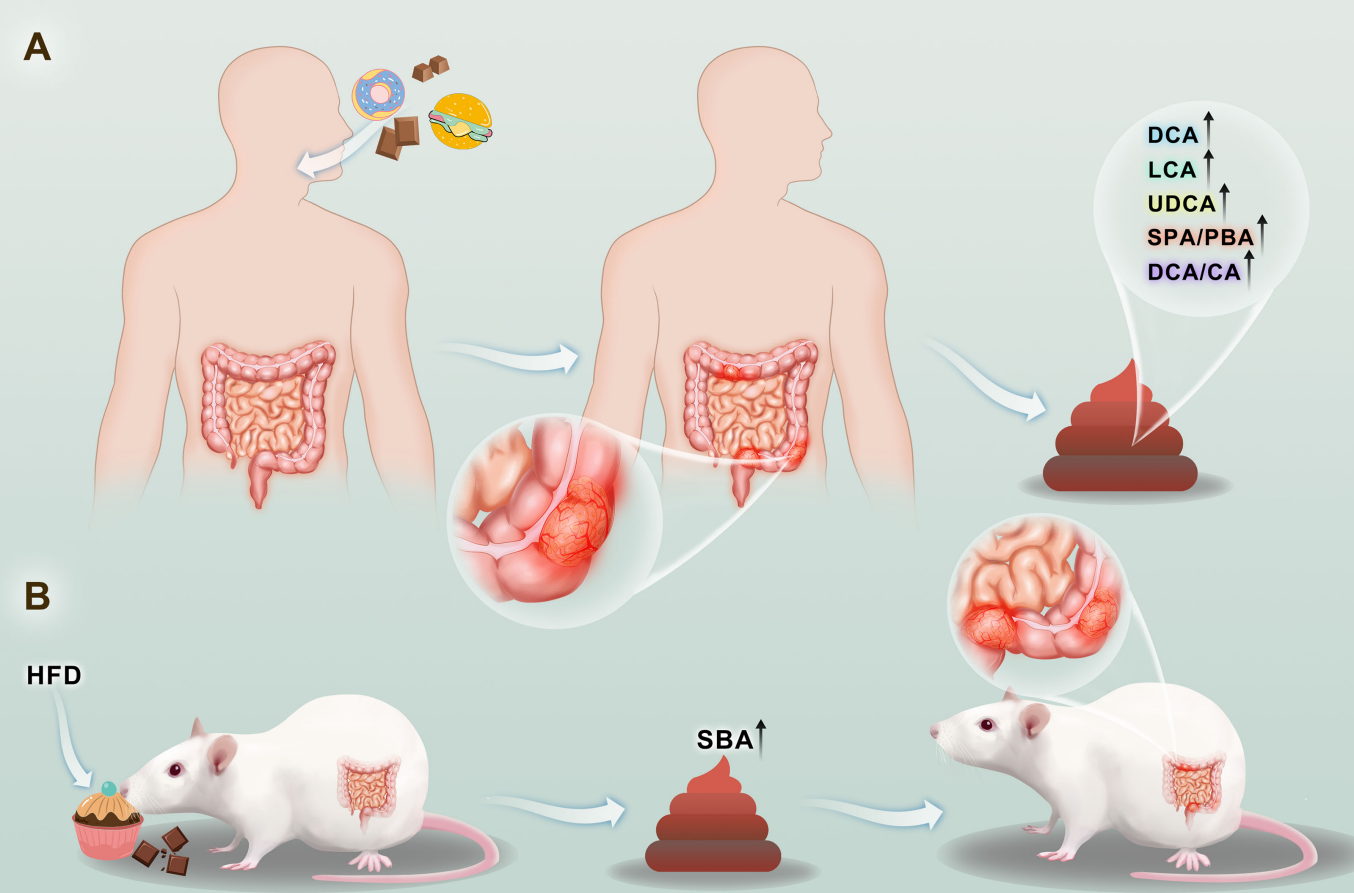
The occurrence and development of colorectal cancer will be accompanied by the increase of secondary bile acid content in stool **(A)**. Consumption of a high-fat diet increases the levels of secondary bile acids in the stool, and increased levels of secondary bile acids have been contributed to the development of colorectal cancer **(B)**.

Epidemiological data showed that the incidence of CRC is also higher in patients with higher fecal bile acid (FBA) concentrations. Due to the rich diet of fat and protein, the incidence of FBA in the feces and CRC in European and American populations is significantly higher than that in Asian and African populations whose diet contains less fat and protein and is rich in cellulose (10). In all of these populations, the concentration of FBA and the incidence of CRC in meat-eating individuals are also higher than those in vegetarian individuals (11). Multiple studies (12) have revealed that the concentrations of DCA, LCA, UDCA, and other indicators in the feces of CRC patients were higher than those in the normal control group, while PBA (CA, CDCA) levels were not different from that of the normal control group (**Figure 1A**).This indicated that the concentration of secondary bile acid increased in the carnivorous individuals, but the concentration of PBA did not change significantly.

These findings show that in our country, high secondary bile acid (SBA) levels in CRC patients may be found as animal experiments have shown that giving a high-fat diet increases the concentration of animal waste FBA and also increases bowel cancer rates; directly increasing the experimental animal intestinal bile acid concentration intake also increases the rate of CRC, indicating that a link between diet and FBA levels and CRC exists, and that FBA is a cause of large intestine cancer rather than a result of the disease (**Figure 1B**) (13). Laboratory studies have shown that bile acids, especially SBA, have a wide range of biological toxicities, such as mutagenesis, cell turnover, cytolysis, and DNA damage (12). Animal experiments also revealed proved that SBAs, in particular their roles in promoting tumorigenesis and as auxiliary carcinogens, have more potent effects than PBAs. Therefore, the SBA/PBA and DCA/CA indices help determine risk (14). LCA/DCA is another effective index for risk evaluation of CRC colon cancer patients in whom the LCA/DCA is higher than that of normal controls, with further illustrates that LCA, rather than DCA, has an auxiliary effect that cause cancer (15).

SBAs mainly include DCA, LCA, and a small amount of UDCA (UDCA), the levels of which are closely related to the occurrence of CRC, especially DCA, which is considered to be a carcinogenic and carcinogenic factor. The occurrence of CRC is a multigene, multistep and multipathway process involving chromosome instability, gene mutation, mismatch modification gene inactivation, and multiple cell signal transduction pathway abnormalities. A high-fat diet and gallbladder disease can increase the content of secondary bile acids in the intestinal cavity, especially the level of DCA, thereby promoting the occurrence and development of CRC.

On the other hand, some reports have confirmed that DCA can also participate in intestinal metabolism to maintain intestinal immune balance. Campbell, Clarissa et al. (16) found that SBA 3β-hydroxy-deoxycholic acid (isoDCA) attenuated the immune stimulating properties of dendritic cells (DCS) by acting on them, thereby increasing Foxp3 induction. This study further reveals the effect of secondary metabolites of bile acids on Tregs and demonstrates that this effect is mediated by antigen-presenting cells. It was also proved that isoDCA limited the activity of FXR in DC and played an anti-inflammatory role, but the role of other bile acid receptors in this process remains to be explored.

**Figure 2:**
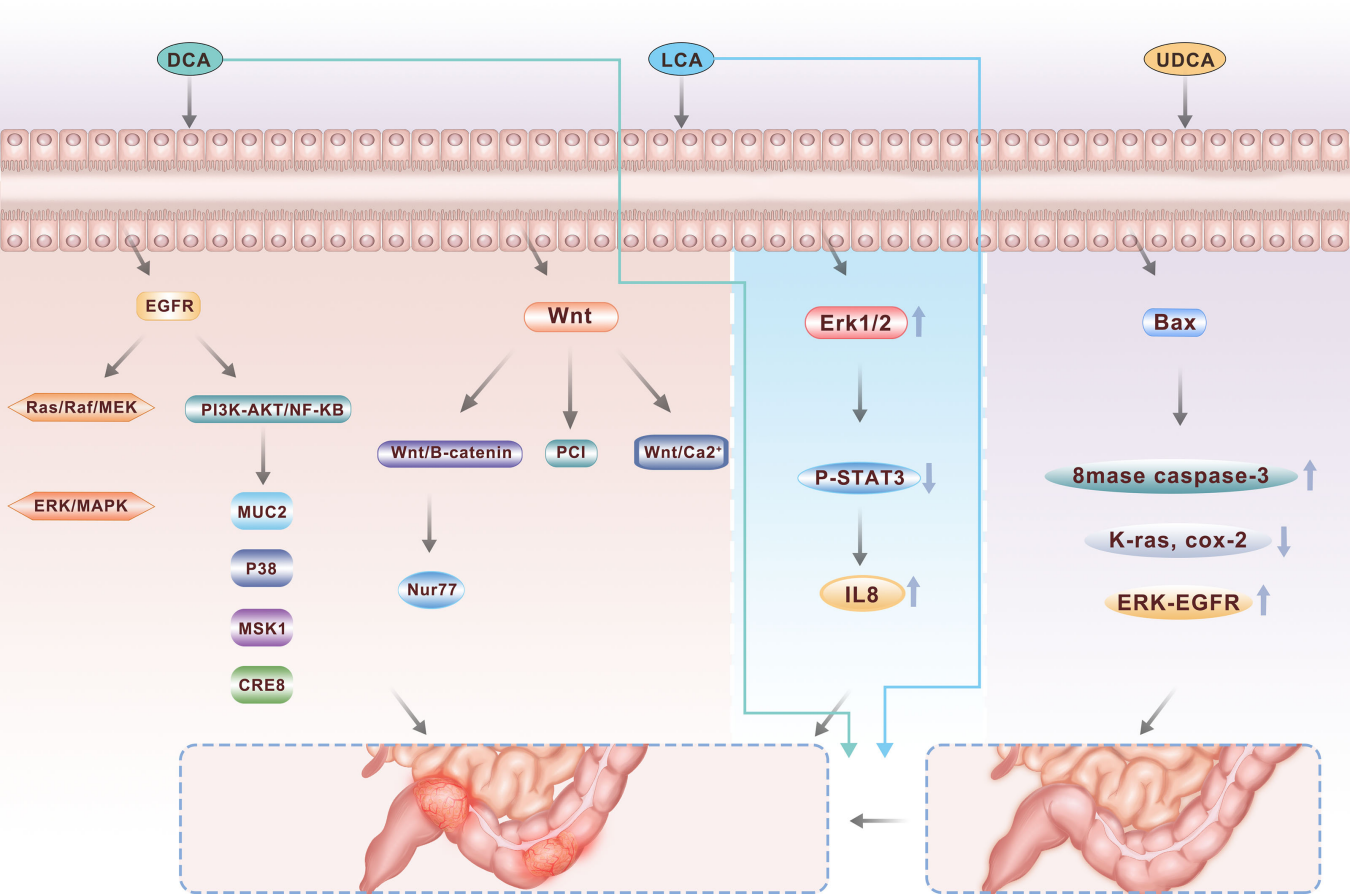
As major secondary bile acids, DCA and LCA are two important pathogenic factors in the occurrence of colorectal cancer, activating multiple signaling pathways including PI3K-AKT, NFkB and Wnt, while UDCA, another component, can play a protective role to a certain extent.

With the development of research, we gradually found that not all bile acids have a role in promoting CRC. Many experiments have shown that during the development of CRC, high concentrations of SBAs play a role in tumor promotion (**Figure 2**) (17).

## Secondary Bile Acid - Deoxycholic Acid

### Deoxycholic Acid Leads to Genomic Instability

DCA causes genomic instability, and DCA-induced genomic instability arises *via* several mechanisms, including oxidative damage to DNA, damage to mitochondria and the endoplasmic reticulum, an increase in the micronucleus rate, and chromosome aneuploid mutations (18). The mechanism is as follows: DCA-induced DNA oxidative damage occurs after cell exposure to high concentrations long-term as a result of nitro DCA and oxidation and induces apoptosis or DNA damage. DNA damage in cells continues to occur in long-term high concentrations of DCA, leading to mutation and natural selection of mutated cells, promoting the development of cancer cells (19). In addition, DCA can cause the dysfunction of several DNA mismatch repair enzymes, such as APC and TP53, by inducing mutations. The disruption of DNA mismatch repair function results in genome microsatellite instability (20). Continuous exposure of intestinal epithelial cells to a high concentration of SBA can induce the production of reactive oxygen species(ROS) and active nitrogen species and induce DNA damage in intestinal epithelial cells through oxidative stress, which can damage genomic stability and increase gene mutations (21). The main manifestations of genomic instability are the appearance of heteroploidy, intrachromosomal instability and gene point mutations (22). Defective repair of DNA oxidative damage induced by DCA increases the risk of CRC. Additionally, a high concentration of litcholic acid can also induce the destruction of DNA molecules and inhibit DNA repair enzymes (23).

The damage induced by DCA in the mitochondria and endoplasmic reticulum is caused by mitochondrial damage and produces excessive ROS (ROS). The production of excessive ROS releases Ca ions from the ER membrane through the activation of inositol triphosphate and alanine receptors, which damages the ER membrane. ER membrane damage leads to impaired protein folding, and the production of excessive ROS exceeds the antioxidant capacity of the cell, leading to the death of colonic epithelial cells (21). Wachs et al. demonstrated that DCA induced apoptosis by changing the permeability of the mitochondrial membrane, independent of the CD95 receptor (24). Many scholars have found that DCA, through NAD(P)H oxidase (which produces protein kinase B, breaking down the original activated protein kinase, and activates p38 and downstream transcription factors to activate proteins in the nucleus and activate AP) and endogenous nitrogen oxygen stress induced by PLA2 can cause genetic damage that changes DNA repair protein structure and can also activate nf-kappa B, an antiapoptotic gene that causes mitochondrial damage and affects autophagy. This effect alters the genetic stability of normal colon cells, the mutant cells selectively proliferate, and the cells become cancerous through the chain reaction of aneuploidy, gene mutation and proliferation (25). Schlottman et al. found that DCA acts on a several colon cancer cell lines in a concentration-dependent and time-dependent manner and induces apoptosis in a short period of time but causes uncontrolled cell proliferation and apoptosis inhibition over a long period of time, disrupting the balance between proliferation and apoptosis and eventually causing loss of homeostasis leading to malignant transformation (26). In addition, Rial et al. treated HCT116 and ht29 cell lines with DCA and found that the expression of CXCL8 increased, and the combination of CXCL8 increase with ap-1 and NF-B activity enhanced cell invasiveness (27).

There are two mechanisms by which DCA induces an increase in the micronucleus rate and chromosome aneuploid mutation: (1) chromosomal deletion and translocation through the instability of the centromere and telomere, and (2) the disruption of the gene-controlled process of mitosis, resulting in asymmetric segregation of chromosomes, initiation of autocatalytic karyotypes, and progression to the cancer cell phenotype (28).

DCA induces CRC by causing genomic instability. Existing mechanisms include DNA oxidative damage, mitochondrial and endoplasmic reticulum damage, increased micronucleus rate, and chromosomal aneuploid mutations. However, the research on the relevant mechanism is still relatively simple and needs to be further explored.

## The Effect of Deoxycholic Acid on the Proliferation and Apoptosis of CRC Cells

DCA is a strong promoter in the early stage of tumor formation and can promote the development of CRC through several mechanisms. The mechanism of inducing cell proliferation and apoptosis is as follows: stable and translocated pronucellin through tyrosine phosphorylation enters the nucleus and stimulates the expression of uPA, uPAR and cyclin D1. Tyrosine phosphorylates and decomposes proctin-catenin to induce cell adhesion loss, while uPA/uPAR receptor-mediated proteolytic enzymes degrade the extracellular matrix and induce cell proliferation with cyclin D1/uPA/uPA, thereby improving the growth and progression of colon cancer cells.

Kong et al. found that DCA could cause miRNA dysfunction and play a role in promoting tumor formation. The growth, migration and invasion of colorectal tumor cells were inhibited by the highly expressed miR-199a-5p, and the cell cycline-related protein CAC1 was regulated by miR-199a-5p. DCA can promote tumor formation by inhibiting the effect of miR-199a-5p and/or promoting the increase in CAC1 expression (29). Reduced coenzyme I and II oxidase activation can also induce protein kinase B (Akt), ERKl/2, p38 and downstream transcription factor activation to activate proteins in the nucleus leading to a-1 protein activation (5). Activation protein-1 is a proto-oncogene mainly composed of c-jun and c-fos that can activate transcription factors, cause chain reactions in downstream genes, and play an important role in cell proliferation and tumor progression (30). Caspase is an apoptosis-specific protease that plays a key role in cell apoptosis. Caspase triggers proteolysis of the cell matrix by activating specific signals, thus inducing apoptosis. There are two main pathways for caspase activation, the endogenous pathway and the exogenous pathway. The endogenous pathway is the mitochondrial-dependent pathway. The cellular stress molecules released into the cytoplasm by mitochondrial cytochrome C are associated with apaf-1 in the presence of ATP to enhance the activation of caspase-9 and the cleavage of endogenous substrate PARP, leading to cell apoptosis. The exogenous pathway is the death receptor-mediated signal transduction pathway. After specific binding with the “death ligand”, the death receptor transmits the apoptotic signal from the outside to the inside of the cell, activates caspase-8, and leads to cell apoptosis (31). Interestingly, recent study reported that antagonizing intestinal FXR with T-β-MCA and DCA could induce proliferation and DNA damage in Lgr5+ cells. On the contrary, selective activation of FXR with Fexaramine D (FexD) and Obeticholic acid (OCA) inhibited the abnormal growth of Lgr5+ cells and the progression of CRC (32).

DCA treatment of BCS-TC2 colonic tumor cells results in apoptosis within 30 min to 2 h; the cells separate into single cells, the cell membranes lose symmetry, the chromatin is condensed, the DNA degrades, mitochondrial permeability changes occur, and the Caspase family and antiapoptotic proteins (Bax) are activated, causing an enzymatic cascade that increases the production of ROS, which leads to mitochondrial membrane potential alteration and the release of apoptosis factors within the cell (33).

Donato F proposed that DCA is an endogenous carcinogen that can promote DNA damage and inhibit the expression of the brca-1 gene in the colonic mucosal epithelium, which is responsible for inducing apoptosis and DNA repair. Dianhua Qiao et al. demonstrated that DCA inhibits the P53 gene through the degradation of the P53 protein mediated by protein kinase. P53 is a well-known tumor suppressor gene, and its inhibition can promote tumors (34). Daniela et al. demonstrated that DCA can induce upregulation of c-fos and cox-2 gene expression in L197 colorectal adenoma cells (35). However, the c-fos gene is related to cell proliferation, and the cox-2 gene is a product of tumor cells. The upregulated expression of these two genes indicates that DCA has a carcinogenic effect. The p53 and brca-1 genes are tumor suppressor genes and mismatch repair genes. DCA inhibits both tumor suppressor genes and mismatch repair genes, thereby inducing CRC. Researchers have used DCA to treat the BCS-TC2 cell line and found that cell separation, cell membrane asymmetry, chromatin concentration, DNA degradation, and cell apoptosis occurred within 2 h (33).

At present, the main conclusion of DCA on the proliferation and apoptosis of CRC cells is that DCA further promotes the proliferation of CRC cells and inhibits the apoptosis of CRC cells. However, there is still no new breakthrough in the specific mechanism and further research is needed.

## Specific Signaling Pathways in CRC Induced by Deoxycholic Acid

The EGFR signaling axis is closely related to the proliferation, apoptosis and survival of colonic epithelial cells and plays an important role in maintaining cell homeostasis (36). There are two major classical EGFR pathways: the mitogen-activated protease pathway (Ras/Raf/MEK/ERK/MAPK pathway) and the phosphatidylacyl-3 kinase (PI3K) pathway. DCA can activate the EGFR signaling pathway in colonic epithelial cells. There are two main explanations for this: one is that DCA stimulates the production and release of epidermal growth factor, leading to the promotion of receptor activity; the other is that DCA activates the receptor by interfering with the structure of the cell membrane, and the current study favors the latter (37). Studies have revealed that DCA can induce tyrosine phosphorylation and activate the EGFR signaling pathway of tyrosine kinases in a ligand-dependent manner, activate the Ras/Raf/MEK/ERK/MAPK pathway, and activate ap-1 to mediate cell proliferation and differentiation (38). DCA can also activate the PI3K/Akt/i-b/NF-B pathway, regulate downstream target molecules such as the caspase family and NF-B transcription factor, thus regulating cell proliferation and apoptosis (39, 40). It has also been found that DCA can promote the transcription of MUC2 mucin in HM3 colon cancer cells by active ting multiple pathways, including the EGFR/PKC/Ras/rf-1/MEK1/ERK/CREB, PI3K/Akt/IkappaB/NF-B and p38/MSK1/CREB pathways, and this effect can be inhibited by the erJNK/c-jun/ap-1 pathway. These results demonstrate a new molecular mechanism for bile acid regulation through the mucin gene, which may help further explain the carcinogenic effect of DCA. In addition, Zhu et al. (41). found that DCA can induce and activate cox-2 through the PKC pathway, and the activation of cox-2, especially in the matrix which is dominated by cancer-related fibroblasts, can significantly increase the proliferation and invasiveness of CRC cells.

The signaling proteins of the Wnt family are involved in several developmental processes during embryogenesis and are involved in tissue homeostasis in adults. The Wnt signaling pathway is pleiotropic and is involved in promoting mitosis, determining cell fate, and differentiation. There are three Wnt pathways, the classical Wnt/b-catenin pathway, the PCP pathway and the Wnt/Ca2+ pathway. Current research focuses on the classical Wnt pathway, that is, that Wnt regulates the expression of b-catenin protein through its Frizzled receptor, thereby regulating the transcriptional expression of downstream genes and ultimately affecting the cell cycle pathway (42). After the treatment of colon cancer cells with a low concentration of DCA, the b-catenin signaling pathway was found to be significantly activated, which improved the proliferation and invasion capabilities of colon cancer cells (43). Nur77 is an immediate early response gene that is rapidly induced by a variety of extracellular stimuli. Increased expression of Nur77 was found in 9 of 12 studied colon tumor tissue samples, and DCA can significantly induce the expression of Nur77 through the Wnt/b-catenin signaling pathway and subsequently promote the growth and migration of CRC cells (44). Using an “Apc min/+” mouse model of sporadic adenomas, 0.2% DCA was added to the drinking water for 12 weeks. DCA increased the “min/Apc +” diversity of the intestinal tumors in the mice and increased the incidence of intestinal adenocarcinoma, probably through enhanced Wnt signaling pathways that stimulate the proliferation of tumor cells and reduce tumor cell apoptosis (45). Litcholic acid also has a similar effect when used to treat normal human colonic epithelial cells and induces the production of colon cancer stem cells, possibly by regulating the M3R and Wnt/13-catenin signaling pathways (46).

The specific signaling pathways induced by DCA in CRC mainly include two categories –EGFR signaling axis and Wnt signaling pathway. DCA induces CRC by activating these two major signaling pathways. Related studies involve cell and animal levels, providing reliable information for us to understand the occurrence and development of DCA and CRC.

## The Effect of Ursodeoxycholic Acid on CRC

Bile acids in the intestinal tract play an important self-regulating role in the regeneration and differentiation of the mucosal epithelium of the large intestine. UDCA may reduce chemical-induced carcinogenesis. UDCA has been found to be chemoprophylactic for colon cancer in patients with primary sclerosing cholangitis (47). Two to seven percent of these patients have ulcerative colitis (UC), which increases the risk of colon cancer three to five times. Two retrospective studies showed that ursoDCA could be used to improve liver function in patients with PSC and UC, and the risk of the development of inflammation-related tumors was reduced compared with that in the nontreatment group. Some studies have suggested that total fecal bile acid (TFBA), DCA, LCA, DCA/CA, LCA/DCA and other indicators in the feces of CRC patients were higher than those in the normal control group (P< 0.01), while primary bile acid(PBA) levels (CA, CDCA) were the same as that in the normal control group (P> 0.05) (48). The relative risk of colon dysplasia and cancer was 0.26 in those who received UDCA, and UDCA significantly reduced the risk of colon dysplasia in UC patients. In contrast, UDCA combated the toxic effects of DCA on colon cells. After pretreatment of HCT116 cells with UDCA and subsequent exposure to DCA, DCA-induced apoptotic cells were significantly reduced (49). K-ras is an oncogene in CRC. UDCA can inhibit the mutation of the k-ras gene and the activation of the wild-type k-ras gene. Additionally, UDCA can inhibit the cox-2 gene through k-ras gene-dependent and k-ras-independent pathways. Thus, UDCA has a positive inhibitory effect on colorectal tumorigenesis (50).

Long-term oral administration of UDCA did not increase the incidence of CRC but showed a decreasing trend. Animal studies have shown that UDCA significantly reduces the number and volume of colon cancer cells induced by carcinogenic agents such as azomethane oxide (AOM). In patients with UC and primary sclerosing cholangitis, the incidence of atypical hyperplasia and carcinoma of the colon decreased after UDCA administration. After administration of UDCA, the concentration of UDCA in the bile increased significantly and the level of DCA in the feces decreased, while UDCA inhibited colonic epithelial hyperplasia. The mechanism may be inhibition of cancer cells by the activation of the apoptotic gene bax, which activates the activity of basic spatholinase (8mase) and caspase-3 (caspase-3), inhibiting the mutation of k-ras and the expression of ras-dependent cox-2, and inhibiting the activation of erk, fra-1 and epidermal growth factor receptor (egfr) caused by DCA. The UDCA concentration in normal bile is very low. Therefore, whether UDCA can inhibit cancer in normal bile metabolism should be further studied.

Cell-level studies have shown that UDCA can induce the apoptosis of CRC cells in the early stage, while DCA mainly induces the apoptosis and necrosis of normal intestinal cells. Therefore, bile acids play an important role in regulating the survival and death of colon cancer cells. UDCA is a hydrophilic bile acid that exists as a protective component of cells. It prevents a variety of stressors, including DCA-induced apoptosis, and its role in the colon was demonstrated by the molecular mechanism of UDCA against the effects of DCA. Pretreatment with UDCA can reduce the occurrence of apoptosis after DCA exposure. UDCA can also significantly affect the activity of neurophospholipase (smase), affecting cell proliferation and colon cell apoptosis depending on the cell conditions (51, 52). The toxicity, growth effect, proliferation process and cell cycle effect of UDCA has been studied in HT29 human colorectal adenocarcinoma cells. UDCA reduced the proliferation rate of HT29 cells. The UDCA rat model shows that UDCA is chemically protective against colon cancer (53) and may reduce the proliferation of colorectal mucosa, but an analysis of the effect of UDCA on human colorectal mucosa revealed that 6 months of UDCA therapy did not appear to change patient adenomas with colorectal mucosa proliferation; thus, the chemical protective effect of UDCA presumably does not occur through modulation of colorectal mucosa proliferation.

In Laurie Barclay’s double-blind study (54), 1285 patients who underwent colorectal adenoma resection in the preceding 6 months were randomized to the UDCA or placebo groups for 3 years or until follow-up colonoscopy. Compared with the placebo, UDCA treatment significantly reduced the recurrence of highly stunted adenomas. Injury resulting from high hypoplasia readily produces colon tumor, and the ability of cells to resist invasion from outside carcinogenic factors is weak. Therefore, UDCA can be considered for treating different pathological diseases based on the different biological mechanisms of tumorigenesis.

Zhang, Huan et al. (55) explored the relationship between UDCA and YAP (Yes Associated Protein) in CRC.UDCA suppressed YAP signaling by activating the membrane G-protein-coupled bile acid receptor (TGR5). TGR5 mainly regulated cAMP/PKA signaling pathway to inhibit RhoA activity, thereby suppressing YAP signaling. Moreover, the restoration of YAP expression alleviated the inhibitory effect of UDCA on CRC cell proliferation. In AOM/DSS-induced CRC model, UDCA inhibited tumor growth in a concentration-dependent manner and decreased expression of YAP and Ki67. UDCA plays a distinguished role in regulating YAP signaling and CRC growth from the PBAs and partial SBAs, demonstrating the importance of maintaining normal intestinal bile acid metabolism in cancer patients. It also presents a potential therapeutic intervention for CRC.

## Effect of Litcholic Acid on CRC

PBAs (cholic acid and chenodeoxycholic acid) are synthesized in the liver and excreted into the duodenum, where they facilitate the absorption of dietary lipids. A small volume of bile acids escapes the enterohepatic circulation and enters the colon; these bile acids are extensively metabolized by bacterial flora to form the SBAs DCA and LCA (56). Substantial evidence implicates DCA and LCA as endogenous cancer promoters (57). Feeding DCA to AKR/J mice, which are genetically resistant to the colon carcinogen azoxymethane (AOM), resulted in a significantly higher level of aberrant crypt foci compared to that in AOM-exposed mice on a control diet (58). Consistent with these observations, a recent human study demonstrated a strong association between low colonic short-chain fatty acid and high bile acid levels in populations at high risk for colon cancer (59) (This article is protected by copyright. All rights reserved 4). LCA induces cell invasiveness by upregulating the expression of the urokinase type plasminogen activator receptor in CRC cells (57). Another study also confirmed that VDR is activated by LCA and participates in the detoxification of toxic bile acids (60).

DCA has been demonstrated to stimulate MAPK signaling through epidermal growth factor receptor activation and calcium signaling in HT-29 colon cancer cells (61). LCA has been found to damage the epithelial lining of the gastrointestinal tract through ROS production, which results in resistance to apoptotic cell death and increased cell proliferation in gastrointestinal tract compartments (62).

IL-8 has been detected in CRC and contributes to poor patient prognosis. However, the effect of LCA on IL-8 expression is still undefined. LCA treatment induces IL-8 expression in CRC HCT116 cells. The results of pharmacological inhibition and mutagenesis studies indicated that Erk1/2 is critical for LCA-induced IL-8 expression. Furthermore, LCA reduces the phosphorylation of STAT3, and the STAT3 inhibitor Stattic accelerates LCA-induced IL-8 expression, suggesting that STAT3 is involved in LCA-induced IL-8 expression. Activation of Erk1/2 functions as an upstream signal of STAT3 suppression induced by LCA. In conclusion, LCA activates Erk1/2 and in turn suppresses STAT3 phosphorylation to induce IL-8 expression in HCT116 cells, thus stimulating endothelial cell proliferation and tube-like formation.

## Bile Acids and Intestinal Flora

The intestinal flora is the largest and most important microecosystem in the human body. Healthy adult intestines contain more than 500 species of bacteria from 30 genera, including 1014 bacteria with a weight of approximately 1000 g and a total genome content that is 100 times greater than that of humans (63). These bacterial genomes are collectively referred to as the “intestinal metagenome” and the “second human genome” and influence human health. The bacteria of the intestinal flora include aerobes, facultative anaerobes and anaerobes and most are obligate anaerobes or facultative anaerobes. Bacteroidetes and Firmicutes account for more than 90% of the intestinal flora (64).

The intestinal flora of the human body is large and versatile. These bacteria can form a biological barrier in the intestinal tract through the placeholding effect, nutrition competition, and several secreted metabolites (65), which can reduce low-level inflammation in the body and protect the integrity of the intestinal wall. Stimulation of the gut to build an effective immune defense system can regulate the absorption and transformation of sugar and fat in the intestine, improve glucose tolerance and oxidative stress, and reduce blood sugar. The intestinal flora can produce binding bile acid hydrolase that converts binding bile acid into free bile acid and reduces intestinal absorption, inhibit the FXR/FGF15 signaling pathway to enhance the activity of CYP7A1 and promote the cholesterol synthesis of bile acid, promote the production of cholesterol oxidase to inhibit the activity of liver fat synthetase, ferment carbohydrates to produce short-chain fatty acids and play the role of lipid regulation.

Intestinal bacteria interact with bile acids, which also affect the composition of the intestinal flora. Bile acids promote the growth of bile acid-metabolizing bacteria and inhibit the proliferation of bile acid-sensitive bacteria (66). Bile duct obstruction prevents bile from entering the intestine, which leads to excessive bacterial proliferation and even translocation in the small intestine, suggesting that bile acids affect the number of intestinal bacteria (67). When mice were given 1.25-5.00 mmol/kg bile acid orally, the proportion of Firmicutes in the intestinal flora increased from 54% to 94% to 98%, while the proportion of Bacteroides and Actinomycetes decreased significantly (68). Bile acids can also stimulate the expression and secretion of inducible nitric oxide synthase and interleukin-18 through the action of FXR, thereby inhibiting the proliferation of intestinal bacteria (69, 70). It has been found that the number of Firmicutes in the intestinal tract of mice with defective bile acid receptor FXR genes is significantly increased, while the number of Bacteroidetes is significantly decreased, as a result of increased bile acid secretion when the FXR receptor is defective, suggesting that bile acid can affect intestinal flora composition through the FXR signaling pathway.

Bile acid is an antibacterial substance that can inhibit the growth and proliferation of bacteria. Bile acids can cause morphological changes in *Helicobacter pylori* and inhibit its growth and proliferation (71). Proteomic studies have shown that both human and porcine bile acids can stimulate the stress response of *Helicobacter pylori*, causing changes in its membrane permeability, interfering with its energy metabolism, and affecting its virulence and colonization ability (72). Oral bile acid significantly inhibited the excessive proliferation of intestinal pathogenic bacteria in model rats with colonic ulcers, inhibited the translocation of pathogenic bacteria, reduced the concentration of serum endotoxin, and prolonged the survival of the model rats (73, 74). The effect of bile acid on *Staphylococcus aureus* has been studied, and it was found that bile acid caused morphological changes in the bacteria, significantly reduced intracellular pH, damaged the integrity of the cell membrane, caused the disappearance of the cell membrane potential, and induced bacterial death. The molecular mechanism by which bile acids inhibit the growth and proliferation of intestinal pathogens is still unclear.

PBA molecules are soluble in water and fat and can interact with and destroy the phospholipid bilayer, causing cell membrane rupture, leading to cell death (75). The conjugated bile acid can freely diffuse through the cell’s outer membrane into gram-negative bacterial cells, cause a cellular stress response, induce cell RNA secondary structure formation, or lead to intracellular protein denaturation inactivation, thus inhibiting bacterial proliferation (76). D'Aldebert et al. (77) found that bile juice acid can induce intestinal epithelial cells to regulate protein kinase (extracellular protein kinase, ERK) 1/2 signaling pathways, activate nuclear triggers of the vitamin D receptor system (VDR) protein, and stimulate the expression and creation of antimicrobial peptide cathelicidin; this group also found that the antibacterial peptides play bacteriostatic roles. Bile acids can damage the DNA of *E. coli*, and *Salmonella*, causing DNA point mutations, frameshift mutations, and even chromosome rearrangement, thus inhibiting bacterial proliferation and inducing bacterial death (78, 79). Proteomics technology, such as that used in Leverrier’s study, revealed that bile acids enter bacterial cells *via* active surface molecules, causing the degradation and loss of function of heat stress shock proteins such as molecular chaperones, and disruption of new protein synthesis and folding, resulting in death. YouYan noted that in addition to bile acids and bacteria, Fe2+ and Ca2+ divalent cations affect physiological functions, including bacterial movement, cell division, gene expression and chemotaxis, thereby inhibiting bacterial proliferation (74).

To resist the damaging effect of bile acids, some intestinal bacteria have adapted to evolution and can resist bile acids. Some pathogens even use bile acids as small molecule inducers to regulate the expression of virulence- or infection-related genes and promote their localization and infection in the intestinal tract. The type III secretion system (T3SS) is an important virulence factor of deputy hemolytic Vibrio transcription regulatory proteins (Vibrio parahaemolyticus transcriptional regulatory proteins, Vtr +) A and C; two transcription factors form the barrel complex and this complex was identified as free bile acid that activates the downstream VtrB, which in combination with its promoter sequences, activates T3SS transcription, induces the expression of downstream virulence factors, and enhances the virulence of the pathogen (80, 81).

Deoxycholate can be used as a small molecular inducer to induce the *Campylobacter* invasion antigen (Cia) promoter and upregulate the expression of Cia, thus greatly improving the infection capacity of *Campylobacter* in epithelial cells (82). In a further study, Kreuder et al. (83) found that bile acids can induce the upregulation of *Campylobacter jejunum* toxicity-related and colonization-related factors and stimulate the upregulation of the expression of 7 noncoding RNAs. Intestinal bile acids can activate the *Clostridium difficile* bacteria spore receptor (*Clostridium difficile* germination - specific protease, CspC) leading to spore germination and *Clostridium difficile* that are capable of pathogenic infection (84). Pathogenic *Escherichia coli* flagella protein gene expression significantly enhanced by bile acids, which induced the expression of adhesion proteins and, subsequently, iron-related proteins, leading to an increase in iron uptake, thus enhancing *E. coli* infectivity (85).

## High-Fat Diet Affects Bile Acid Metabolism and Destroys the Integrity of the Intestinal Barrier

The intake of a high-fat diet can stimulate the secretion of cholecystokinin and pancreatin, cause contraction of the gallbladder and common bile duct, increase the release of bile, and cause a significant increase in FXR expression in the jejunum and tumor necrosis factor-α (TNF-α) expression in the colon, thus altering the metabolism of bile acid (86). As the main component of bile, bile acid is converted from PBA to SBA by intestinal symbiotic bacteria. SBAs are hydrophobic and cytotoxic to colonic crypt epithelial cells; DCA may be involved in the activation of protein kinase C (PKC) and the destruction of the cell membrane. DCA has an antiapoptotic effect in most cells and can induce the proliferation of colon epithelial cells and adenoma cells. Compared with normal conditions, the content of several bile acids in the colon, especially DCA, is increased in CRC patients, while the proportion of UDCA is decreased. The proportion of bile acids in feces also changed similarly, and the content of DCA in the feces of CRC patients was higher than that in the feces of healthy people. Normal colonic epithelial cells and adenoma cells are continuously exposed to high concentrations of highly hydrophobic bile acids and resist apoptosis induced by hydrophilic or low concentrations of hydrophobic bile acids, such as UDCA, and overactivate the EGFR/MAPK signaling pathway, resulting in irreparable oxidative DNA damage of intestinal epithelial cells and destroying the intestinal barrier to induce CRC (87). In addition, the ability of bile acids to destroy the phospholipid bilayer provides antibacterial properties (68), which cause changes in the intestinal flora and increases the number of harmful bacteria. Therefore, a high-fat diet can increase the content of bile acid in CRC patients, further affecting the proliferation and apoptosis of colonic epithelial cells, destroying the intestinal barrier, changing the intestinal flora, and promoting the occurrence of CRC.

The intestinal epithelium and the substances it secretes are the first barrier against the invasion of bacteria and other pathogens. A decrease in intestinal barrier function and the thinning of the mucus layer leads to the infiltration and transfusion of harmful bacterial products such as lipopolysaccharides (LPS), which leads to a systemic inflammatory response. The normal human body sustains intestinal injury when absorbing fat, which is repaired after 50 min (88), while long-term excessive fat intake can activate mast cells in the intestinal mucosa, cause them to secrete TNF-α, IL-1β, IL-4 and IL-13 and other regulatory factors, and indirectly increase intestinal cell permeability, which allows more LPS to penetrate the blood (especially through intercellular infiltration), causes endotoxemia and stimulates the inflammatory response. In addition, the increase in TNF levels resulting from the phosphorylation of myosin light chain kinase may lead to contraction of the cytoskeleton and rupture of tight junctions. The low expression of tight junction proteins such as claudin-1, claudin-3, occludin and junction adhesion molecule-1 in the intestinal mucosa of high-fat diet animals also supports the view that a high-fat diet destroys the intestinal barrier (89). It has also been suggested that a high-fat diet and obesity can lead to inflammatory changes in the ileum and proximal colon of mice and change intestinal permeability through the activation of focal NF-κB and the expression of TNF-α and IL-1β. Recently, it has been found that the process by which a high-fat diet changes intestinal barrier function is dynamic and differs across locations. The early stage of a high-fat diet can produce a rapid but reversible increase in intercellular permeability in the ileum of mice followed by a compensatory increase in the tight junction protein ZO-1, while intracellular permeability is increased in the early stages after colon removal. In addition, there was a continuous and gradual increase in cross-cell permeability and a decrease in the number of goblet cells (90). The depletion of eosinophils in the intestines of mice fed a high-fat diet was also associated with an increase in intestinal permeability (91). In addition, a high-fat diet can affect the density and function of intestinal endocrine cells (such as Pan’s cells and enterochromaffin cells), and too much fat intake can reduce the number of Pan’s cells (92) and reduce intestinal resistance to bacteria, thus promoting the occurrence of intestinal tumors.

It has been recognized that chronic low-grade inflammation is closely related to the occurrence and development of CRC. Markers of chronic inflammation, such as C-reactive protein (CRP), in the blood have a certain suggestive effect on the risk for CRC (93). In recent years, studies have suggested that a high-fat diet can increase body weight, subcutaneous fat, visceral fat and the size of fat cells (94) and cause metabolic disorders such as obesity and insulin resistance, which alter the function of immune cells, cytokines and other immune regulators to promote the occurrence and development of chronic low-grade inflammation in the body and promote CRC. Macrophage aggregation, soluble regulatory factors secreted by adipocytes and neutrophil activity play a key role in the inflammatory response induced by a high-fat diet (95). Adipose tissue releases free fatty acids and adipocytokines, such as leptin, TNF-α, IL-6, CRP, plasminogen activator inhibitor-1 (PAI-1) and monocyte chemoattractant protein-1 (MCP-1). MCP-1 can attract circulating monocytes, activate the phenotypic transformation of M1 macrophages, and influence the levels of TNF-α, IL-1β and IL-6 secreted by macrophages. Inflammatory factors, such as ROS and rnos, rapidly accumulate in the infection site of colonic epithelial cells, causing apoptosis with a subsequent increase in peripheral neutrophils. The increase in TGF-β secretion can also promote the malignant transformation of epithelial cells (95). In a mouse tumor model, a high-fat diet upregulated the expression of COX-2, PGE2, cyclin D1, and PCNA, increased the expression of NF-κB, p65 and β-catenin, and inhibited p21 CIP1/WAF1 by increasing histone deacetylase (HDAC). In addition, the MAPK/ERK and PI3K/Akt/mTOR signaling pathways were activated, which eventually led to a series of cascade reactions, including epithelial interstitial (EMT) and inflammatory reactions, to promote tumor development (96). The above series of inflammatory reactions leads to the disordered growth of colorectal epithelial cells and ultimately promotes the occurrence and development of CRC.

The combination of a high-fat diet and K-ras gene mutation can cause a change in the intestinal bacterial population, which is related to the weakening of Pan’s antibacterial defense, which subsequently blocks the recruitment of dendritic cells and major histocompatibility complex II (major histocompatibility complex II)-positive cells in the gut-associated lymphoid tissues (GALTS). The expression of MHC-II promotes the occurrence and development of CRC (97). A high-fat diet can increase the expression of TNF-α mRNA in the ileum of SPF mice, activate the NF-κB EGFP pathway in intestinal epithelial cells, immune cells and endothelial cells, and increase the number of lipopolysaccharide-producing bacteria to induce excessive LPS entry into the circulatory system, resulting in endotoxemia and promoting the intestinal inflammatory response, insulin resistance and other metabolic abnormalities. The above changes also occur in GF mice fed SPF fecal bacteria after exposure to a high-fat diet, suggesting that a high-fat diet influences intestinal flora, affects intestinal barrier function and induces an inflammatory response (89). High-sugar, high-fat and high-calorie diets can rapidly affect the intestinal flora and lead to weight gain. Transplanting the fecal bacteria of non-obese mice into obese mice can reverse this effect, and the weight of the mice can be reduced. Fecal bacteria from healthy donors are not transplanted into human patients on a high-fat diet; however, if other methods, such as digestive tract diversion operations, are adopted, the level of *Sclerotinia* will be increased. If the proportion of *Enterococcus* returns to normal, weight loss can be achieved, and metabolic disorders such as obesity can be relieved (98). In addition to the significant change in the proportion of *Sclerotinia* to *Enterococcus*, a high-fat diet also led to an increase in *Rikenellaceae, Ruminococcuceae*, proteobacteria, archaea, *Clostridia*, *Prevotellaceae* and rod-like bacteria. The number of bacteria was decreased, and the change in intestinal flora upregulated iNOS and COX-2 expression, activated NF-κB through the TLR4 signaling pathway, and promoted the secretion of inflammatory cytokines to stimulate the inflammatory response (99). Recent studies have suggested that a decrease in enterobacteria may lead to a decrease in IL-10 and an increase in intestinal permeability in the early stage of excessive fat intake (68). In addition, in an inflammatory environment, a high-fat diet can increase the number of *E. coli*, reduce the thickness of the mucus layer, increase intestinal permeability, upregulate NOD2 and TLR5 expression and promote the secretion of TNF-α. These changes enhance the colonization ability of adherent invasive *E. coli* in CRC, thus further aggravating inflammation and promoting the occurrence of CRC (100).

Therefore, various studies have shown that a high-fat diet will affect the metabolism of bile acids and destroy the integrity of the intestinal barrier, thus promoting the occurrence and development of CRC.

## Conclusion

In summary, bile acids increase the risk for CRC, and the mechanism and influencing factors of bile acids on CRC development are complex, including influencing the proliferation and apoptosis of colonic epithelial cells, destroying the intestinal barrier to increase intestinal permeability, and changing the intestinal flora. Research and exploration of the above mechanisms can provide a reference for the prevention and treatment of CRC in the future. The application of nonsteroidal anti-inflammatory drugs, probiotics and their products, lifestyle changes and other means may become effective interventions. We look forward to further research and exploration in the future to provide a further theoretical basis for the occurrence and development of CRC and new treatment directions.

## Author Contributions

HX and GJ contributed to the conceptualization of this paper and made the frame. YL and SZ contributed to original draft preparation. DH participated in part of the text arrangement and image conception. WZ, GJ, and HX revised the manuscript. All authors contributed to the article and approved the submitted version.

## Funding

This work was supported by National Nature Science Foundation of China, No. 81620108030, 81874206, 82104466; Shanghai Frontier Research Base of Disease and Syndrome Biology of Inflammatory cancer transformation, No. 2021KJ03-12; Shanghai Rising-Star Program, No. 20QA1409300; and the Program for Young Eastern Scholar at Shanghai Institutions of Higher Learning, No. QD2019034. Artificial Intelligence Cooperation project of Xuhui District Science and Technology Commission, No. 2020-004.

## Conflict of Interest

The authors declare that the research was conducted in the absence of any commercial or financial relationships that could be construed as a potential conflict of interest.

The reviewer WC declared a shared affiliation with the authors to the handling editor at time of review.

## Publisher’s Note

All claims expressed in this article are solely those of the authors and do not necessarily represent those of their affiliated organizations, or those of the publisher, the editors and the reviewers. Any product that may be evaluated in this article, or claim that may be made by its manufacturer, is not guaranteed or endorsed by the publisher.

## Abbreviations

CRC, Colorectal cancer; FXRs, farnesol X receptors; CYP7A1, cholesterol 7α-hydroxylase; PBA, primary bile acid; SBA, secondary bile acid; CA, cholic acid; CYP8B1, sterol‐12α‐hydroxylase; CYP27A1, mitochondrial sterol‐27‐hydroxylase; CDCA, chenodeoxycholic acid; CYP7B1, oxysterol‐7a‐hydroxylase; TCA, taurocholic acid; GCA, glycine cholic acid; TCDCA, taurine chenodeoxycholic acid; GCDCA, glycine chenodeoxylate oxycholic acid; BSH, bile salt hydrolases; DCA, deoxycholic acid; LCA, lithocholic acid; UDCA, ursodeoxycholic acid; PI3K, phosphatidylacyl-3 kinase; ROS, reactive oxygen species; UC, ulcerative colitis; TFBA, total fecal bile acid; AOM, azomethane oxide; EGFR, epidermal growth factor receptor; VDR, vitamin D nuclear receptor; Cia, *Campylobacter invasion antigen;* TNF-α, tumor necrosis factor-α; PKC, protein kinase C; LPS, lipopolysaccharides; CRP, C-reactive protein; PAI-1, plasminogen activator inhibitor-1; MCP-1, monocyte chemoattractant protein-1; HDAC, histone deacetylase; EMT, epithelial interstitial; GALTS, gut-associated lymphoid tissues.
